# Prognostic significance of microRNA 17–92 cluster expression in Egyptian chronic lymphocytic leukemia patients

**DOI:** 10.1186/s43046-021-00097-x

**Published:** 2021-12-06

**Authors:** M. M. Khalifa, N. E. Zaki, A. A. Nazier, M. A. Moussa, R. Abdel Haleem, M. A. Rabie, A. R. Mansour

**Affiliations:** 1 Department of Internal Medicine, Hematology Unit, Faculty of Medicine, Alexandria University, Alexandria, Egypt; 2 Department of Clinical Pathology, Faculty of Medicine, Alexandria University, Alexandria, Egypt; 3 Department of Medical Laboratory Technology, Pharos University, Alexandria, Egypt

**Keywords:** Chronic lymphocytic leukemia (CLL), Prognosis, MicroRNA, miRNA17-92 cluster

## Abstract

**Background:**

Abnormal expression patterns of microRNAs (miRs) play an important role in the development and progression of malignancy. Identification of the clinical significance and prognostic value of these small molecules in chronic lymphocytic leukemia (CLL); a disease of heterogeneous biological landscape and clinical course, has always been of tremendous translational value.

**Aim:**

To evaluate the prognostic value of microRNA17-92 cluster members in Egyptian CLL patients.

**Methods:**

The expression levels of miR17-92 cluster members were evaluated by qRT-PCR, including miR17, miR18a, miR19a, miR19b-1, miR20a, and miR92a-1. Other investigations included serum LDH, serum β2 microglobulin (β2M), CD38 and ZAP70 expression by flow cytometry, fluorescence in situ hybridization (FISH) for 17p deletion, and imaging studies (computerized tomography (CT) scans of neck, chest, abdomen, and pelvis or PET-CT scans).

**Results:**

Overexpression of all members of the miRNA17-92 cluster was detected in CLL patients compared to controls (*p* =  < 0.001 for all miRs while *p* = 0.01 for miR19b-1). A significant positive correlation between Hb and miR17 and a significant negative correlation between Hb and miR19b-1 were observed (*p* = 0.041, 0.017 respectively). A statistically significant positive correlation between miR19b-1 expression and each of the WBCs and absolute lymphocytic count (ALC) was detected (*p* = 0.023, 0.022 respectively). Moreover, a statistically significant relation between miR19b-1 expression and advanced Binet stages was also found (*p* = 0.05). Regarding miR18a, a statistically significant positive correlation with LDH level was found (*p* = 0.003). We also found a significant positive correlation between miR92a-1 and β2M level (*p* = 0.005), as well as a significant relation between miR17 and negative CD38 expression (*p* = 0.034). However, no significant relationships between any of studied miRNA expression levels and 17p deletion or response to treatment were observed. Patients who expressed miR19b-1 were significantly indicated to start therapy at diagnosis (*p* = 0.05). The overall survival of CLL patients included in our study was 90.2% after 1 year from the time of diagnosis. Patients with high expression of miR19a had better OS than those with low expression (*p* = 0.04).

**Conclusions:**

Overexpression of all members of the miR17-92 cluster was detected in Egyptian CLL patients. MiR18a, miR19b-1, and miR92a-1 also have an adverse prognostic value while miR17 can be considered a good prognostic marker. High expression of miR19a is associated with better OS.

## Background

Chronic lymphocytic leukemia (CLL) is a malignancy characterized by clonal proliferation and accumulation of mature, CD5 positive B lymphocytes in the blood, marrow, and secondary lymphoid tissues [1]. CLL represents the most common leukemia in the Western world; it accounts for 30–40% of all adult leukemias. CLL is primarily a disease of the elderly with a median age of diagnosis in European populations of around 70 [2]. 40–60% of patients diagnosed with CLL are asymptomatic diagnosed accidentally during a routine medical examination; the remaining patients may present with constitutional manifestations (weakness, fatigue, night sweats, and fever), lymphadenopathy, splenomegaly, and maybe infections or autoimmune diseases [3].

Diagnosis of CLL requires the presence of ≥ 5 × 10^9^/L clonal B lymphocytes in the peripheral blood, sustained for at least 3 months confirmed by demonstrating immunoglobulin light chain restriction using flow cytometry [4]. CLL is a heterogeneous disease in terms of biological landscape with a variable clinical course. The well-established prognostic biomarkers in CLL include host factors (i.e., gender and age), disease markers [i.e., lymph node involvement (size, site), hepatomegaly, splenomegaly, lymphocyte doubling time (LDT), WBC count, absolute lymphocytic count (ALC), anemia, thrombocytopenia, Rai and Binet staging], antigen expression (i.e., CD38, ZAP70, and CD49d/VLA-4), serology (i.e., LDH, β2M, thymidine kinase, and IL-8), genetics (i.e., del 17p and TP53 gene mutation, del 11q, del 13q, trisomy 12, complex karyotype), epigenetics (DNA methylation), molecular markers (NOTCH1 mutation, ATM, SF3B1 mutation, BIRC3 mutation, BRAF mutation), and immunogenetics (i.e., IGHV gene mutational status and BCR structure) [5].

Several parameters should be considered before recommending treatment for CLL including clinical stage, patient's symptoms, fitness, comorbidities particularly potential organ toxicity of the newer targeted agents, genetic risk, and treatment availability [2]. Allogeneic stem cell transplantation has been considered the treatment of choice for high-risk CLL patients and is the only curative approach although its role is decreasing in the era of novel agents including the BTK, PI3K, and BCL2 inhibitors [6]. Minimal residual disease assessment improves prediction of outcome in patients with CLL [7].

MicroRNAs (miRs) are a family of small, non-coding RNAs that regulate the expression of target messenger RNAs at the post-transcriptional level. MiRs are extensively involved in proliferation, apoptosis, development, and hematopoietic lineage differentiation and their deregulation appears to play a vital role in the onset, progression, and dissemination of many cancers [8]. Expression profiling revealed that microRNA signatures can distinguish normal B cells from malignant CLL cells and are associated with prognosis, progression, drug resistance, and BCR stimulation. Associations between miRs and chromosomal abnormalities suggest that miRs may be involved in the pathogenesis of CLL. MiR15-16 cluster, miR34b/c, miR29, miR181b, miR17-92, and miR155 family members are the most deregulated microRNAs in CLL [9].

The polycistronic miRNA17-92 cluster is located in the non-protein-coding gene at 13q31. The precursor transcript derived from the miR-17–92 gene contains six tandem-loop hairpin structures that ultimately yield the six mature miRNAs miR17, miR18a, miR19a, miR19b-1, miR20a, and miR92a-1 [10]. As a strong oncogene, miR-17–92 regulates multiple cellular processes that favor malignant transformation, promoting cell survival, rapid cell proliferation, and increased angiogenesis [11].

In this study, we aimed to identify the expression and the prognostic implications of miRNA-17–92 cluster in CLL Egyptian patients. Correlations between different members of the cluster with the known prognostic markers of CLL, including 17p deletion, serum LDH, serum β2 microglobulin, and CD38 as well as ZAP70 expression and the stage of the disease at diagnosis were all evaluated. In addition, our study also intended to illustrate the relation between the expression of each member of the miRNA 17–92 cluster and time to start chemotherapy as well as the disease outcome.

## Methods

The present study was conducted on 40 treatment naïve Egyptian patients either newly diagnosed CLL or in the “watch and wait” phase. The patients presented to Alexandria main university hospital and Damanhur oncology center. Twenty healthy age- and sex-matched subjects were also enrolled as controls. The samples were collected over 20 months during the period from between May 2019 and December 2020.

The diagnosis of CLL was established based on the modified International Workshop on CLL (iwCLL) 2018 criteria [4]. A written informed consent was obtained from all study subjects. The approval of the Research Ethics Committee of the Alexandria Faculty of Medicine was obtained prior to the study. All the patients were subjected to history taking focusing on the presence of B symptoms (fever, night sweats, and weight loss), symptoms of anemia, infections, or bleeding episodes, and thorough clinical examination with emphasis on the presence of lymphadenopathy and/or hepatosplenomegaly.

Laboratory investigations included complete blood counts (CBC) that were performed on an ADVIA-2120i automated cell counter (Siemens Healthcare Diagnostics Inc., USA) followed by microscopic examination of peripheral blood smears. Serum LDH level was performed on the Chemistry auto analyzer Dimension RxL Max (Siemens Healthcare Diagnostics, USA). Serum β2 microglobulin was analyzed by auto-analyzer Alegria (Orgentec, Germany).

Immunophenotyping was carried out using Becton–Dickinson FACS Caliber Flow Cytometer equipped with CellQuest Software (BD Biosciences, San Diego, California, USA) for determining the flow cytometric expression of CD38 (clone T-16; Cat# 07,778) and ZAP70 (clone G-4; Cat# 17,760) (Dako Cytomation, Denmark). The threshold of CD38 and ZAP70 positive expression was set at or above 30 and 20% respectively of the gated lymphocytes.

Fluorescence in situ hybridization (FISH) for 17p deletion was performed using Vysis LSI TP53/CEP 17 FISH probe kit (Abbott, Illinois, USA, Cat# 05N56-020).

Quantitative determination of miRNAs expression levels:

Total RNA, including miRNA isolation, was carried out with the miR- Neasy Mini Kit (QIAGEN, Maryland, USA, Cat# 74,004) according to the manufacturer’s instructions. Complementary DNA (cDNA) was synthesized from the purified RNA samples using miScript II RT Kit (QIAGEN, Maryland, USA, Cat# 218,161) according to the manufacturer’s protocol on a CFX96 Bio-Rad PCR system (Singapore). cDNA was stored at – 20 °C until further processed. Expression of miRNAs was carried out using miScript primers assays for miRNA 17, 18a, 19a, 19b-1, 20a, and miRNA 92a-1. (QIAGEN Sciences, Maryland, USA, Cat# 331,231) on a Stratagene Mx3000P QPCR System (Stratagene, Amsterdam, NL, Europe) with miScript SYBR® Green PCR Kit (QIAGEN, Maryland, USA, Cat.#218,073) according to the manufacture instructions. The relative expression of each miRNA was calculated with the comparative CT method (2 − ^ΔΔCT^) after normalization for the expression of SNORD68 as endogenous controls.

Radiological assessment in the form of computerized tomography (CT) scans of neck, chest, abdomen, and pelvis were performed initially for all CLL patients at diagnosis and for follow-up after 6–8 cycles of chemotherapy.

Patients were classified according to the modified Rai and Binet staging systems. For patients who were indicated for therapy, treatment modalities included FCR (fludarabine, cyclophosphamide, and rituximab) and ibrutinib. Response to treatment was assessed according to iwCLL 2018 [4]. The patients were followed up for a period ranging from 6 to 25 months after the enrollment in the study until the end of the study in May 2021.

Overall survival (OS) was estimated for the patients after a period of 12 months of diagnosis and at the end of the study. Also, a comparison between high and low expression of each miR in the cluster in relation to OS was performed.

### Statistical analysis

All statistical analyses were performed using the IBM SPSS software package version 20.0 (Armonk, NY: IBM Corp). Kolmogorov–Smirnov test was used for normality distribution of data. A chi-square test was used for categorical variables, to compare between different groups. Fisher’s exact or Monte Carlo correction was used when more than 20% of the cells have an expected count less than 5. Student’s *t*-test was used for quantitative variables, to compare between two studied groups. The Mann–Whitney test was used for abnormally quantitative variables, to compare between two studied groups. The Kruskal–Wallis test was used to compare more than two groups. Pearson correlation was used to determine the relationship between two quantitative continuous variables. Receiver operating characteristic curve (ROC) was used to plot for miRNAs, the area under the ROC curve denotes the performance and the 95% confidence intervals. Based on these parameters, the diagnostic accuracy of each miRNA was evaluated at a specific Δ*C*_t_ cut-off value detected using Youden’s method for the optimal cut-off, by calculating sensitivity and specificity, accuracy, and positive and negative predictive values. Patients were stratified according to the median value of miRNA into 2 subgroups: the upper 50% of values and the lower 50%. The significance of the obtained results was judged at the 5% level. Overall survival distributions were plotted using Kaplan–Meier estimates. Statistical differences between Kaplan–Meier curves were calculated using the Log-rank test.

## Results

The CLL patients included in the study were 22 males (55%) and 18 females (45%), while the control group were 11 males (55%) and 9 females (45%). The age of patients ranged from 40 to 73 years with a mean of 60 ± 8.77 years, similar to the controls, where the age ranged from 39 to 72 years with a mean of 61 ± 7.58 years. The clinical features of the CLL patients were summarized in Table 1. The laboratory investigations and prognostic markers were summarized in Tables 2 and 3.

**Table 1:** Clinical features in CLL patients

Presenting features	Number of cases (*n* = 40)	Percentage (%)
B symptoms	16	40%
Lymphadenopathy	24	60%
Splenomegaly	21	52.5%
Anemia	11	27.5%
AIHA	3	7.5%
Thrombocytopenia	9	22.5%
ITP	4	10%

Lymphadenopathy alone or with splenomegaly was the most predominant clinical presentation among the patients of the study group followed by B symptoms. Both anemia and thrombocytopenia were present but to a lesser extent

**Table 2:** Comparicon between CLL patients and controls as regards CBC parameters.Hb concentration and platelet count were significantly lower in CLL patients compared to the controls, while WBC and ALC were significantly higher in CLL patients compared to the controls

	**Patients (*n* = 40)**	**Controls (*n* = 20)**	***p* value**
**Hb (g/dL)**			
Mean ± SD	11.10 ± 2.73	13.08 ± 0.73	0.001
Median (min.–max.)	11.25 (3.8–15.6)	13.1 (11.8–14.5)	
**Platelets (× 10**^**9**^**/L)**			
Mean ± SD	174.25 ± 91.44	270.65 ± 83.12	< 0.001
Median (min.–max.)	159 (24–402)	146 (170–422)	
**WBC (× 10**^**9**^**/L)**			
Mean ± SD	95.22 ± 107.10	6.71 ± 1.74	< 0.001
Median (min.–max.)	42.2 (10.39–392)	6.6 (4.2–10)	
**ALC (× 10**^**9**^**/L)**			
Mean ± SD	86.28 ± 105.84	2.79 ± 0.68	< 0.001
Median (min.–max.)	36.4 (6.7–384)	2.91 (1.26–3.57)	

*Hb* hemoglobin, *WBC* white blood cells, *ALC* absolute lymphocyte count; *p* value significant when p ≤ 0.05

**Table 3:** Prognostic parameters in CLL patients. LDH was elevated in 42.5% of patients, whereas β2M was elevated in 77.5% of patients. CD38 positivity was documented in 32.5% of patients, while ZAP70 positivity was reported in 37.5% of patints. 17p del/ p53 mutation was reported in 10% of patients

Prognostic parameter	Number of cases (*n* = 40)	Percentage (%)
**LDH (U/L)**		
Normal	23	57.5%
Elevated	17	42.5%
Mean ± SD	508.12 ± 276.41	
Median (min.–max.)	438.5 (166–1481)	
**β2M (mg/L)**		
Normal	9	22.5%
Elevated	31	77.5%
Mean ± SD	4.8 ± 2.5	
Median (min.–max.)	4.05 (1.7–10.5)	
**CD38**		
Positive	13	32.5%
Negative	27	67.5%
**ZAP70**		
Positive	15	37.5%
Negative	25	62.5%
**17p del/ p53 mutation**		
Positive	4	10%
Negative	36	90%

### Treatment plan and response

At presentation, 17 out of 40 patients (42.5%) were indicated to initiate first-line treatment, while 23 patients were assigned to wait and see strategy and were monitored periodically every 3 months for disease progression. Two of the latter group progressed to more advanced stages and were indicated to start treatment after a period of 5 and 7 months from the time of diagnosis. Complete remission (CR) was achieved in 6 patients (35%), partial remission (PR) in one patient,with an overall response of 41%. Seven patients showed no response; stable disease in 3 patients (17%), and progressive disease in 4 patients (23%). One patient died after the first cycle of chemotherapy while 2 patients were lost for following-up. Four patients died, two of them from sepsis and the other two died because of refractory/progressive disease.

### Results of miR17-92 cluster

**Fig. 1:**
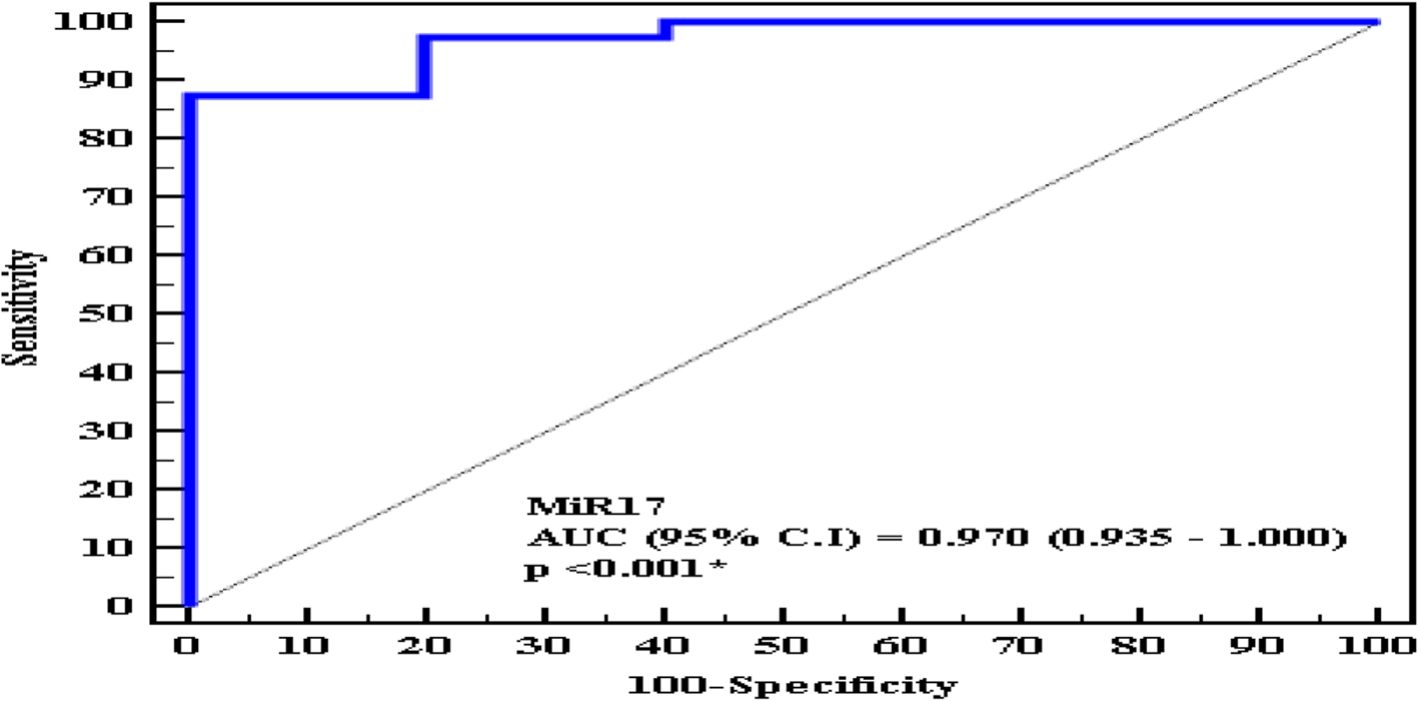
ROC curve for miR17 to discriminate patients (*n* = 40) from control cases (*n* = 20)

**Fig. 2:**
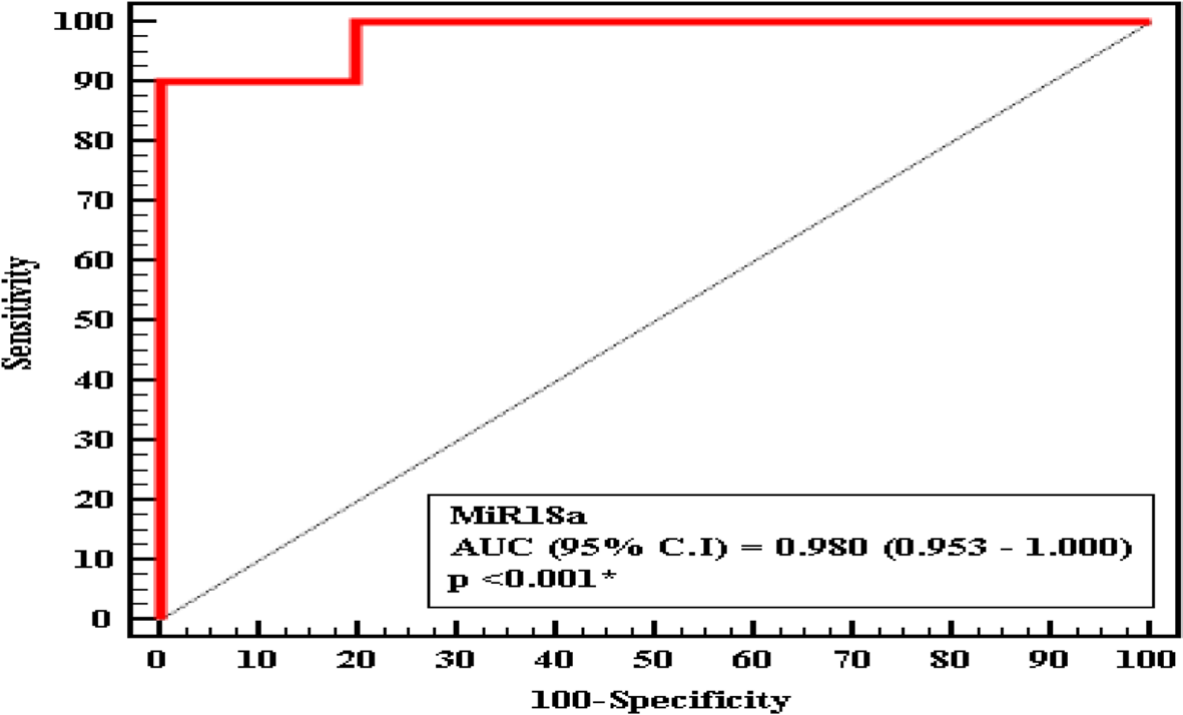
ROC curve for miR18a to discriminate patients (*n* = 40) from control cases (*n* = 20)

**Fig. 3:**
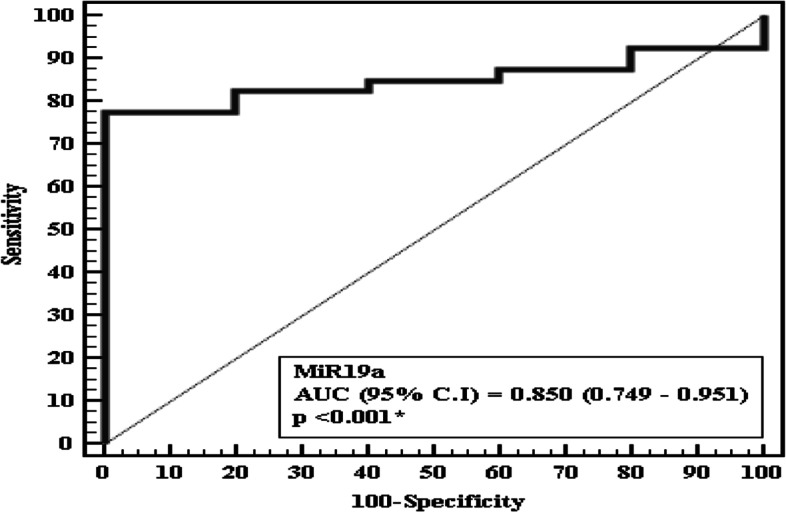
ROC curve for miR19a to discriminate patients (*n* = 40) from control cases (*n* = 20)

**Fig. 4:**
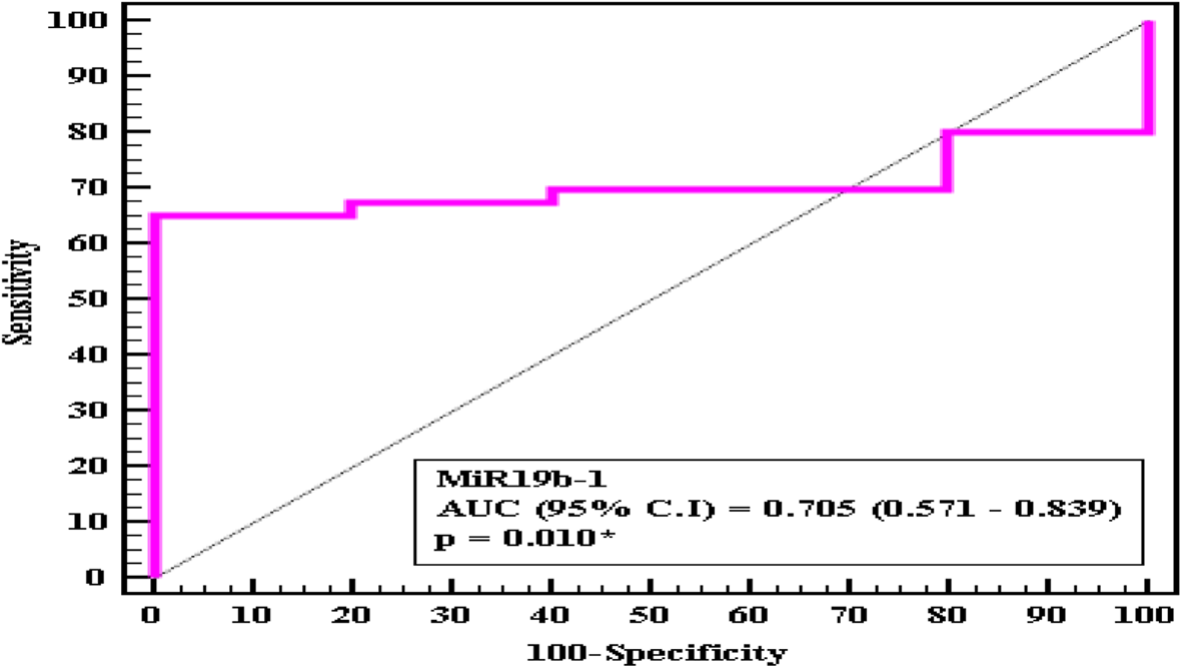
ROC curve for miR19b-1 to discriminate patients (*n* = 40) from control cases (*n* = 20)

**Fig. 5:**
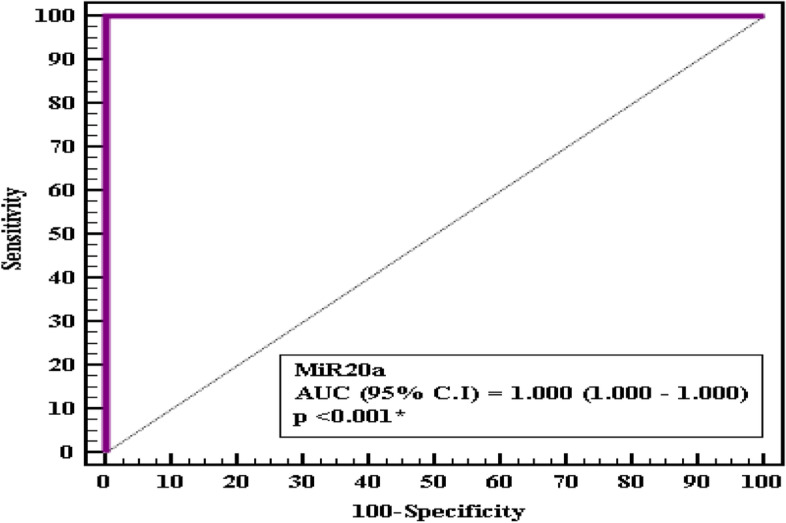
ROC curve for miR20a to discriminate patients (*n* = 40) from control cases (*n* = 20)

**Fig. 6:**
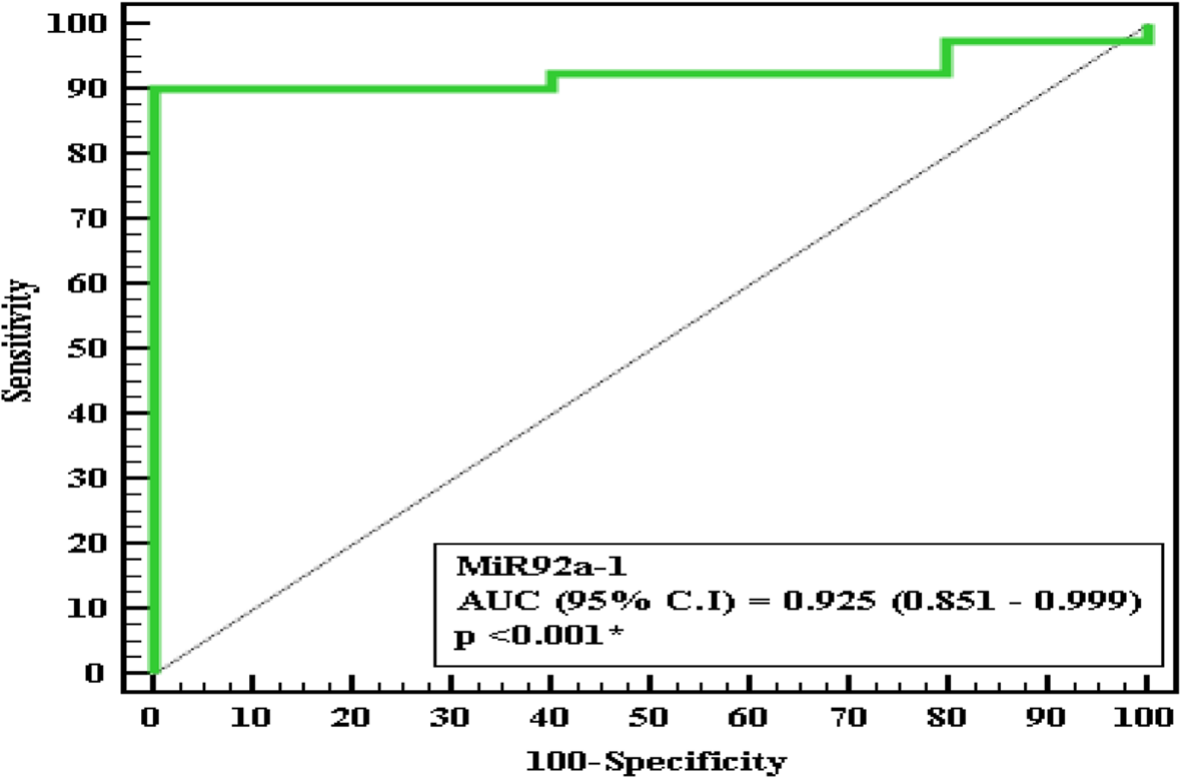
ROC curve for miR92a-1 to discriminate patients (*n* = 40) from control cases (*n* = 20)

Serum levels of all members of the miR17-92 cluster were compared in CLL patients to the control group. Overexpression of all members of the miRNA cluster in CLL patients was detected (Table 5). The diagnostic performance of each miRNA was analyzed with ROC curves and the associated AUC (*p* =  < 0.001 for all miRs while *p* = 0.01 for miR19b-1) (Table 4) (Figs. 1, 2, 3, 4, 5, and 6).

**Table 4:** Distribution of CLL patients among stages of Binet and modified Rai staging systems. According to the modified Rai staging system 16 patients had low-risk disease, 15 patients had intermediate risk and 9 patients were in the high-risk category. According to the Binet staging system, 18 patients (45%) had stage A disease, 13 patients (32.5%) had stage B disease and 9 patients (22.55%) had stage C disease

Clinical stages	Number of cases (*n* = 40)	Percentage (%)
**Binet staging system**		
Stage A	18	45%
Stage B	13	32.5%
Stage C	9	22.5%
**Modified Rai staging system**		
Low-risk	16	40%
Intermediate-risk	15	37.5%
High-risk	9	22.5%

There were no significant differences in any of the miRNA expression levels as regards age.

**Fig. 7:**
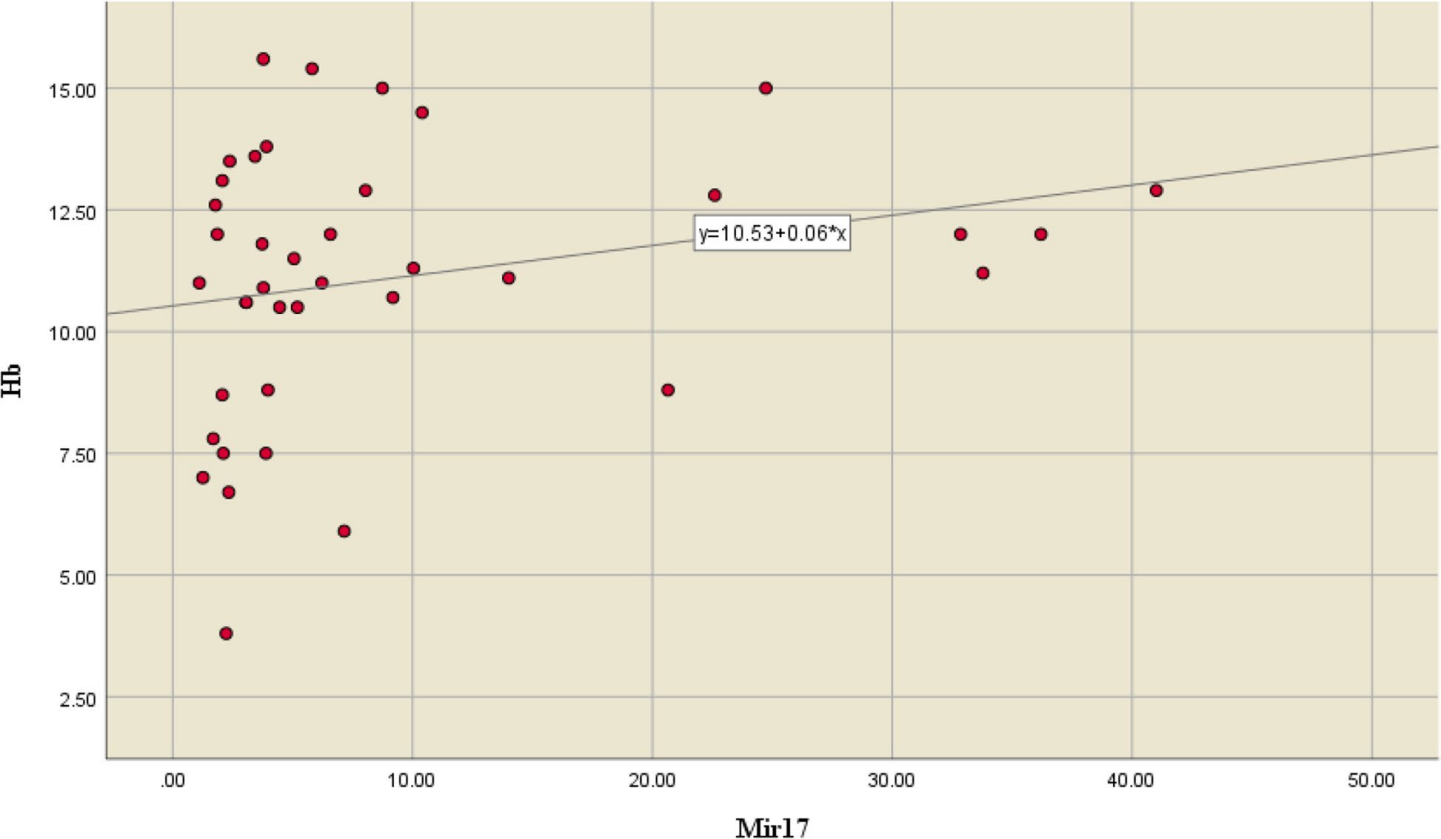
Positive correlation between miR17 expression and hemoglobin concentration in CLL patient (*r* = 0.324, *p* = 0.041)

**Fig. 8:**
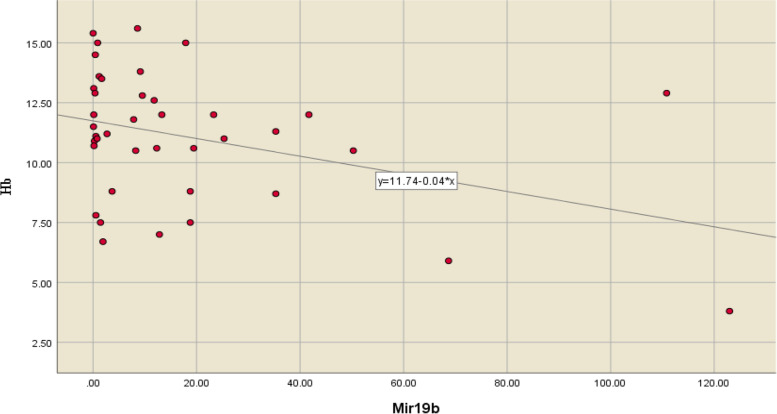
Negative correlation between miR19b-1 expression and hemoglobin concentration in CLL patients (r =  − 0.376, *p* = 0.017)

Correlation studies revealed a statistically significant positive correlation between Hb concentration and miR17 (*p* = 0.041) (Fig. 7) and a statistically significant negative correlation between Hb concentration and miR19b-1 (*p* = 0.017) (Fig. 8).

**Fig. 9:**
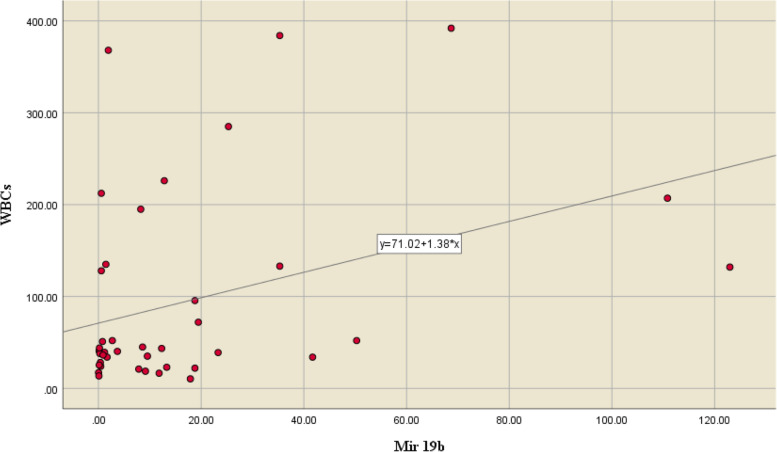
Positive correlation between miR19b-1 expression and WBCs in CLL patients (*r* = 0.359, *p* = 0.023)

**Fig. 10:**
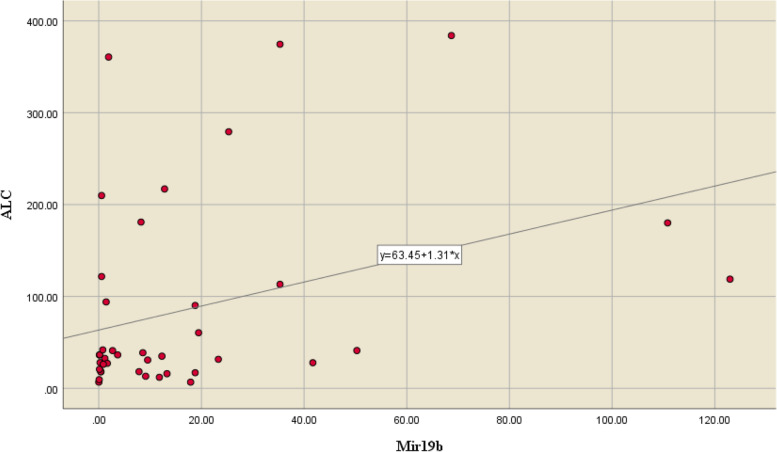
Positive correlation between miR19b-1 expression and ALC in CLL patients (*r* = 0.362, *p* = 0.022)

Also, statistically significant positive correlations were similarly observed between miR19b-1 expression level and each of WBCs and ALC (*p* = 0.023, 0.022 respectively) (Figs. 9 and 10).

However, none of miR17-92 members showed a significant correlation with the platelet count.

Regarding the clinical staging (Table 5), a significant relation was found between miR19b-1 expression and the advanced stage of the Binet system (*p* = 0.05). The same miR was related to the high-risk category of the modified Rai system, although unfortunately, the result did not reach a statistical significance (*p* = 0.061).

**Table 5:** Cases vs controls as regards miRNA cluster. All members of the cluster were overexpressed in the patient group than the control group

Parameter	CLL cases (*n* = 40)	Controls (*n* = 20)	Test of significance (*p* value)
**MiR17**			
Min.–max	1.11–41.01	0.4–2.05	*Z* = − 5.9 (*p* < 0.001)
Mean ± SD	9.16 ± 10.67	1.1 ± 0.5	
Median	4.22	1.06	
**MiR18a**			
Min.–max	1.31–30.78	0.54–2.16	*Z* = − 6 (*p* < 0.001)
Mean ± SD	9.07 ± 7.37	1.1 ± 0.56	
Median	6.8	0.94	
**MiR19a**			
Min.–max	0.09–1189.38	0.55–1.87	*Z* = − 4.4 (*p* < 0.001)
Mean ± SD	89.36 ± 216.41	1.09 ± 0.47	
Median	9.11	0.98	
**MiR19b-1**			
Min.–max	0.02–122.96	0.49–1.5	Z = − 2.5 (*p* = 0.01)
Mean ± SD	17.48 ± 27.87	1.07 ± 0.35	
Median	8.42	1.13	
**MiR20a**			
Min.–max	2.33–53.89	0.62–1.6	*Z* = − 6.3 (*p* < 0.001)
Mean ± SD	10.86 ± 10.82	1.05 ± 0.34	
Median	7.25	1.04	
**MiR92a-1**			
Min.–max	0.55–42.75	0.65—1.4	*Z* = − 5.3 (p < 0.001)
Mean ± SD	6.78 ± 7.93	1.03 ± 0.26	
Median	4.36	0.98	

*Z* Mann–Whitney test; significant (*p* ≤ 0.05)

**Fig. 11:**
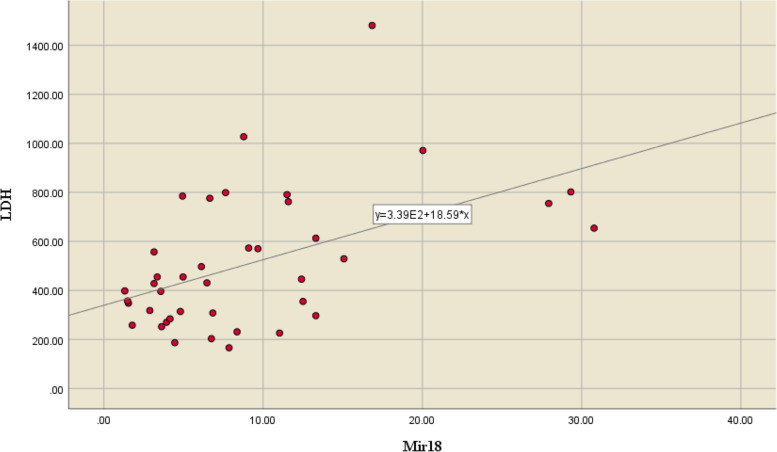
Positive correlation between miR18a expression and serum LDH in CLL patients (*r* = 0.458, *p* = 0.003)

Regarding the relationship between members of the miR 17–92 cluster and different prognostic markers of CLL, miR18a showed a statistically significant positive correlation with LDH (*p* = 0.003) (Fig. 11). Similarly, miR19b-1 showed a positive correlation with LDH, but the results did not reach statistical significance (*p* = 0.0748).

**Fig. 12:**
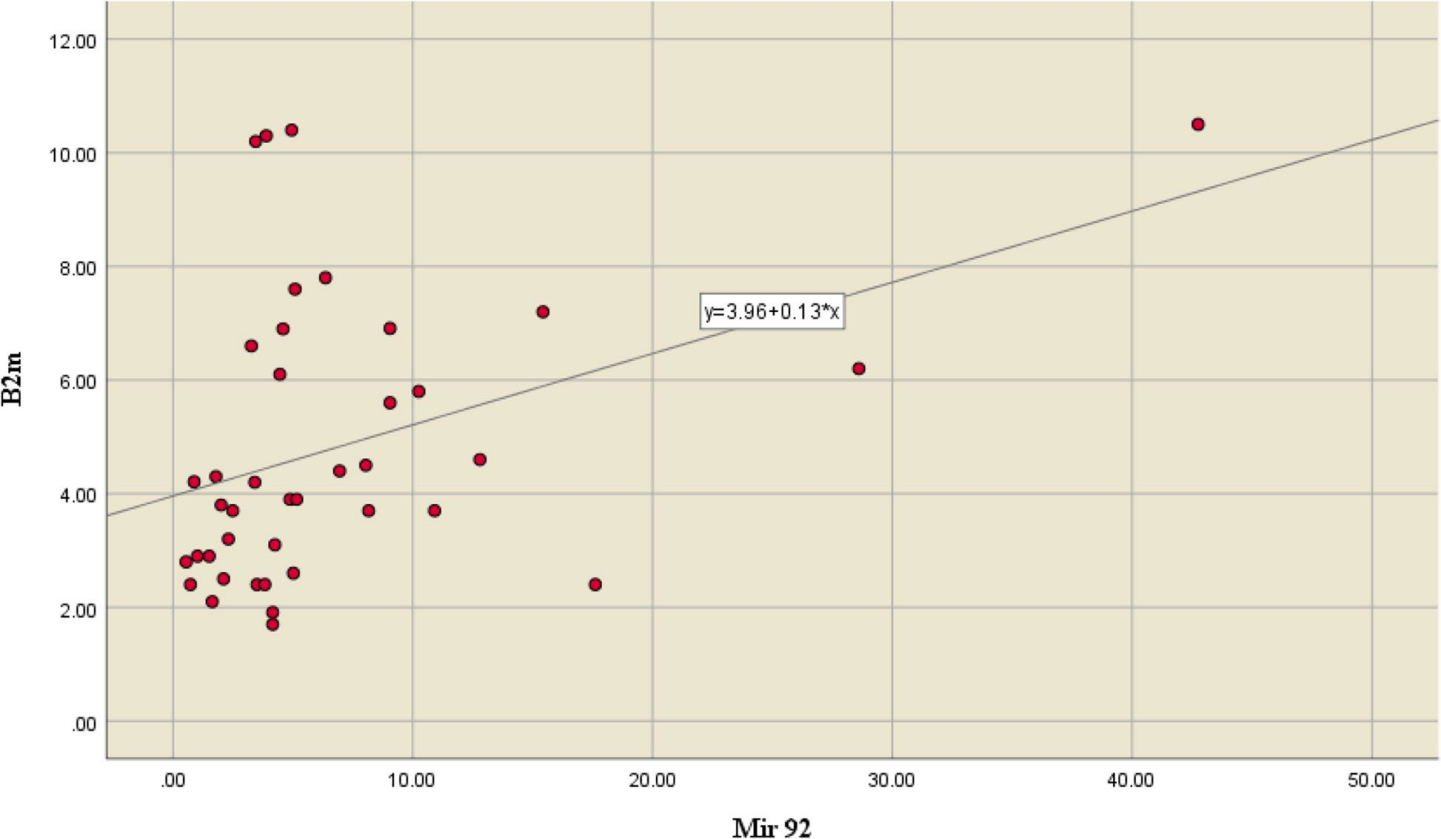
Positive correlation between miR92a-1 and serum β2M in CLL patients (*r* = 0.438, *p* = 0.005)

For β2M, correlation studies revealed a statistically significant positive correlation between miR92a-1 and β2M (*p* = 0.005) (Fig. 12) while the correlation between β2M and either of miR18a (*p* = 0.057) miR19b-1 (*p* = 0.088) did not reach statistically significant levels.

On comparing the studied miRs levels to CD38 expression, negative CD38 was associated with higher expression levels of miR17, and the result reached a statistical significance (*p* = 0.034) (Table 6).

**Table 6:** Relation between miRs and some prognostic markers

	**CD38**			**ZAP70**			**17p del/ p53 mutation**		
	Positive (*n* = 13)	Negative (*n* = 13)	*p* value	Positive (*n* = 15)	Negative (*n* = 25)	*p* value	Positive (*n* = 4)	Negative (*n* = 36)	*p* value
**MiR17**									
**Min.**–**max**	1.11–33.78	1.86–41.01	**0.034**	1.11–32.85	1.26–41.01	0.061	1.26–32.85	1.11–41.01	0.26
**Median**	2.34	6.22		3.07	5.81		2.23	4.76	
**MiR18a**									
**Min.**–**max**	2.88–27.93	1.31–30.78	0.749	1.31–30.78	1.48–29.32	0.091	2.88–11.58	1.31–30.78	0.652
**Median**	6.75	6.84		4.8	8.36		6.65	6.80	
**MiR19a**									
**Min.**–**max**	0.09–190.81	0.43–1189.38	0.333	0.63–1189.38	0.09–190.81	0.41	1.93–189.49	0.09–1189.38	0.718
**Median**	7.76	16.63		6.44	10.45		18.98	9.11	
**MiR19b-1**									
**Min.**–**max**	0.02–122.96	0.13–110.81	0.851	0.13–68.69	0.02–122.96	0.655	1.44–41.70	0.02–122.96	0.652
**Median**	8.59	8.24		3.69	9.53		7.38	8.42	
**MiR20a**									
**Min.**–**max**	2.81–33.64	2.33–53.89	0.073	2.33–53.89	2.81–39.45	0.459	3.54–53.89	2.33–39.45	0.875
**Median**	4.8	9.66		5.82	8.41		5.51	7.87	
**MiR92a-1**									
**Min.**–**max**	0.73–15.43	0.55–42.75	0.762	0.55–28.6	0.73–42.75	0.162	2.49–8.16	0.55––42.75	0.787
**Median**	4.88	4.25		3.41	4.95		3.93	4.36	

For ZAP70 expression, comparison between the studied miRs expression levels and ZAP70 expression, a statistically significant relation between ZAP70 negativity and high expression levels of miR17 above the median in CLL patients (*p* = 0.022) Table 6.

Comparison between 17p deletion/TP53 mutation and the studied miRs expression did not reveal any significant relations Table 6.

Regarding patients’ indication to start chemotherapy, it was found that patients who express miR19b-1 were more likely indicated to start treatment early, and the result was statistically significant (*p* = 0.05). However, no statistically significant relation was documented between any of the studied miRs and response to treatment.

### Survival analysis

**Fig. 13:**
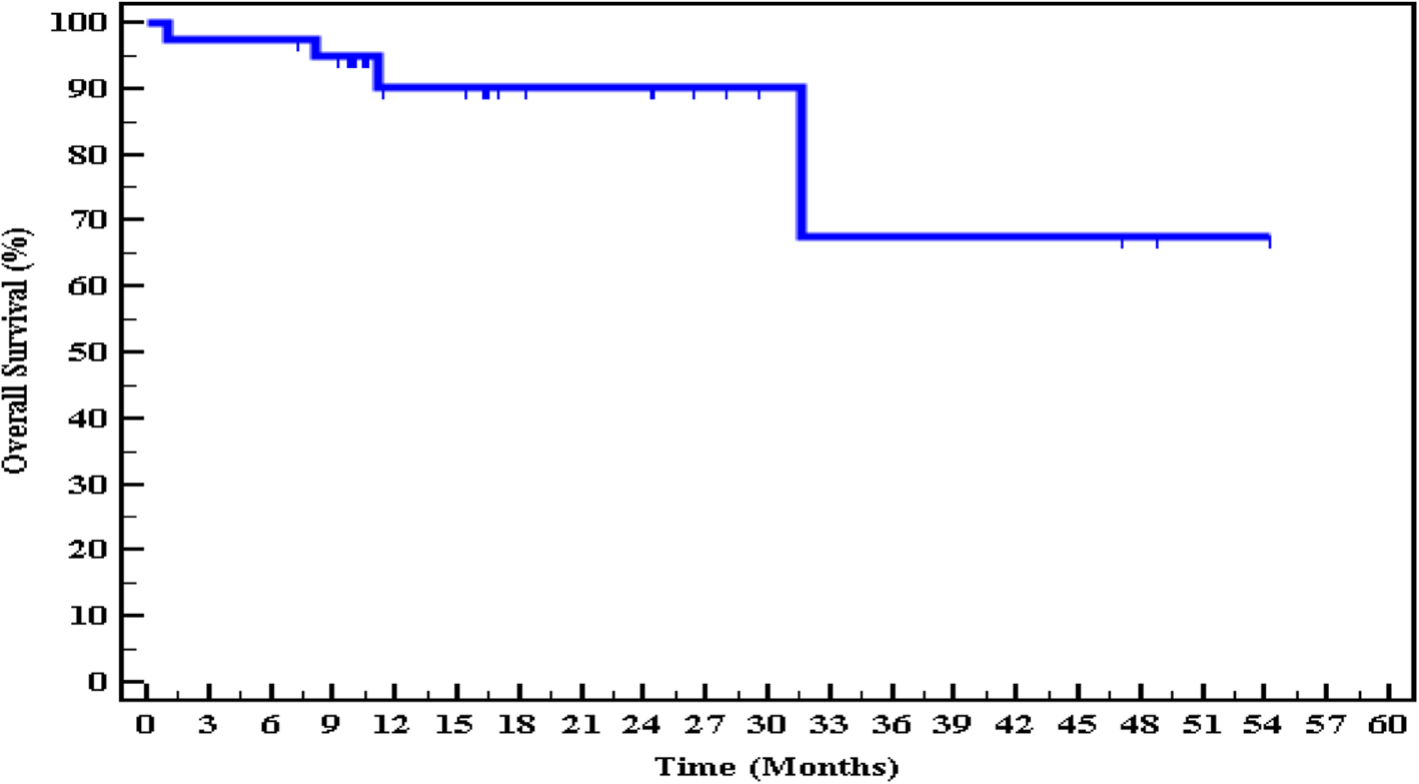
Kaplan–Meier survival curve for overall survival of CLL patients in months

The overall survival of CLL patients included in our study was 90.2% after 1 year from the time of diagnosis, and reached 67.6% by the end of May 2021 with a mean time of follow-up of 40 months (Fig. 13).

**Fig. 14:**
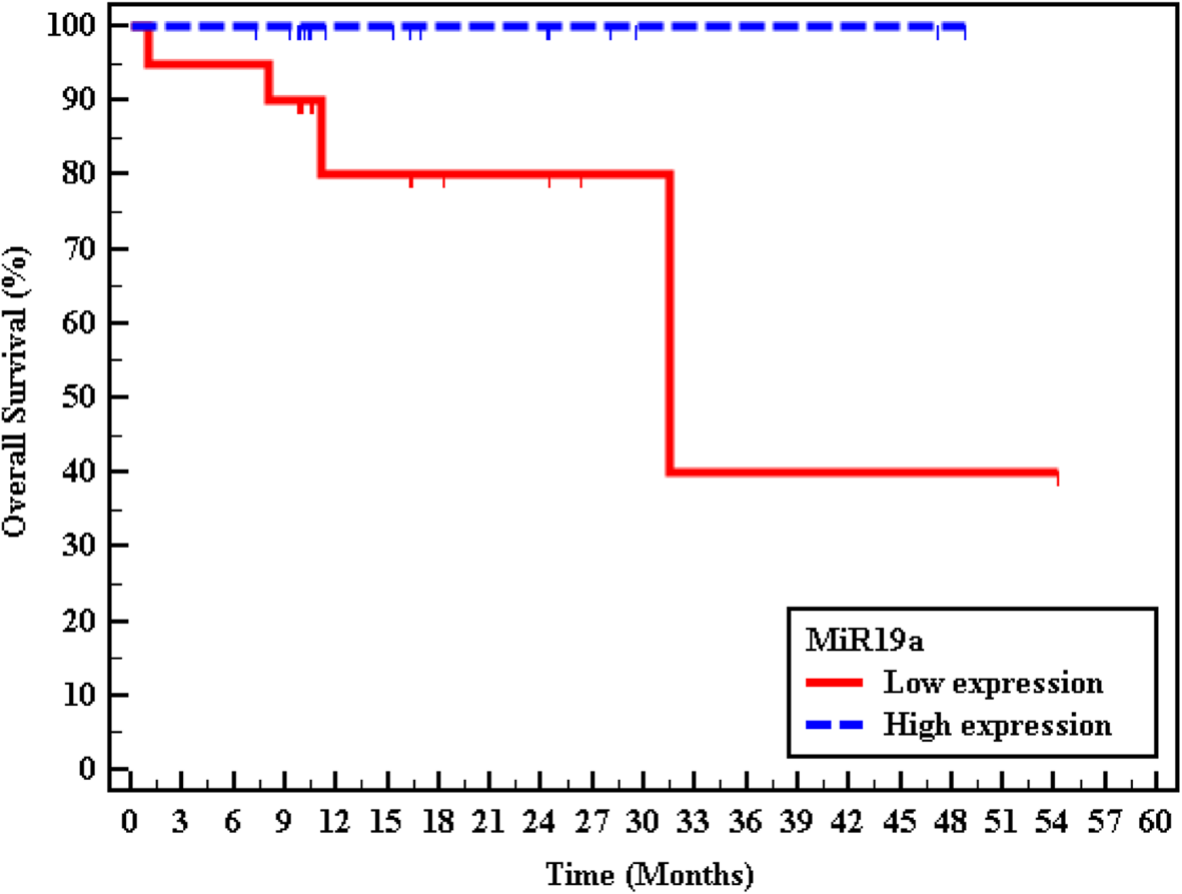
Kaplan–Meier survival curve for overall survival with miR19a. Statistical differences between Kaplan–Meier curves were calculated using the log-rank test. CLL patients with high expression had better OS (*p* = 0.04)

A significant difference in OS was found between CLL patients with different miR19a expression levels. Patients who have high expression of miR19a had abetter OS (*p* = 0.04) (Fig. 14). Otherwise, no other significant differences were demonstrated for the other members of the cluster.

## Discussion

MiR17-92 is one of the best-known polycistronic miRNA clusters. The cluster’s members have fundamental roles in normal development, and dysregulation of their expression leads to numerous diseases including cancers and immune, neurodegenerative and cardiovascular diseases [12]. The miR-17–92 family is among the most famous miRNAs and has been identified as an oncogene, being widely expressed in a high variety of malignancies including both hematological (AML, ALL, CML, NHL, and MM) and non-hematological cancers [13].

The present study revealed that all members of the miRNA17-92 cluster were overexpressed in CLL patients in comparison to the control group, and the differences were statistically significant. This finding may reflect that this cluster plays an important role in the pathogenesis of CLL in Egyptian patients.

The expression levels of members of miR17-92 cluster in CLL vary among different studies. In this study, there was no statistically significant difference between expression levels of all members of the cluster. Similar results were reported by Yan et al. (2019) [14].

In the current work, differential expression as well as correlations between each members of this cluster and different parameters of prognostic influence in CLL were evaluated.

Regarding miR17 appeared to have a good prognostic value in Egyptian CLL patients. This is attributed to the significant positive correlation between miR17 and hemoglobin level, a statistically significant relation between miR17 and CD38 negativity, and a statistically significant relation between ZAP70 negativity and high expression of miR17 above the median in CLL patients. Our findings are contrary to the study done by Culpin et al. (2010) [15] who demonstrated that miR17-5p, a high-risk factor, predicted those patients with low ZAP70 or mutated IGVH were more likely to require earlier treatment which means miR17-. Again, our results are contrary to Bomben et al. (2012) [16] who demonstrated increased miR17 expression in unmutated IGVH and ZAP70 positive cases.

In the current study, miR18a showed a bad prognostic impact. This finding is explained by the statistically significant positive correlation between miR18a and LDH and the positive correlation between miR18a and β2M, though the latter did not reach a statistically significant level. Moreover, miR18a expression was associated with poor response to therapy, yet the correlation did not reach a statistically significant level. This finding coincides with Culpin et al. (2010) [15] who demonstrated that expression of the miR18a was a high-risk factor as it identified the patients with low CD38 who were more likely to progress.

Although miR19a is an oncomiR and its expression level was significantly higher in Egyptian CLL patients, as well as its high expression was associated with better OS. The present study failed to demonstrate any significant relation /correlation between miR19a and the other prognostic markers, clinicalstage, and indication for or response to treatment. A similar result was reported by Farzadfard et al. (2020) [17] where his study demonstrated that miR19a3p is one of the most deregulated miRs in CLL. In addition, they suggested that it can play a role in the pathogenesis of CLL and has a great potential as a diagnostic and therapeutic tool for CLL patients.

In the meantime, miR19b-1 predicts an inferior prognostic value in CLL patients. This is owed to statistically significant positive correlations between miR19b-1 expression and each of WBCs and ALCs, and a significant negative correlation between miR19b-1 expression and hemoglobin concentration. Moreover, we found positive correlations between each of LDH, β2M, and miR19b-1 although these correlations did not reach a statistically significant level. Furthermore, a statistically significant relation between miR19b-1 expression in CLL patients and advanced Binet stages was demonstrated obviously. Similarly, miR19b-1 was slightly related to the high-risk category of the modified RAI system, but this relation was not statistically significant.

Similar results were reported by Olive et al. (2009) [18] who identified miR19 as the key oncogenic component of miR17-92. This is attributed to its role in promoting c-MYC-induced lymphomagenesis by repressing apoptosis, repression of the tumor suppressor PTEN, and activation of the Akt–mTOR (mammalian target of rapamycin) pathway, thereby functionally antagonizing PTEN to promote cell survival.

In this study, no specific microRNA signature was detected among patients who had either autoimmune hemolytic anemia or immune thrombocytopenia. Downregulation of both miR19a and miR20a in patients with CLL subsequently developing AIHA was reported in another study [19].

The present study did not reveal any significant relation /correlation between miR20a and the other prognostic markers, clinical stage, and indication for or response to treatment. On the other hand, a study by Moussay et al. (2011) [20] showed that miR-20a was found to be positively correlated with longer time to treatment in CLL.

In the current work, miR92a-1 exhibited an adverse prognostic impact. This may be demonstrated by a statistically significant positive correlation between miR92a-1 and β2M. In addition, higher expression of miR92a-1 above median value was detected in CLL patients who were eligible for therapy at their presentation compared to those assigned to the wait and see strategy, yet this did not reach a statistically significant level. This is totally contrary to the work of Papageorgiou et al. (2019) [21] who demonstrated lower miR92a-3p expression levels in CLL patients than control individuals while no significant associations were found between miR92a-3p overexpression and CLL patients’ clinical and pathological features. Also, they stated that miR92a-3p positive patients have significantly higher survival rates.

The present study failed to demonstrate any significant relation between miR17-92 expression levels and TP53 mutation. This is contrary to Li et al. (2012) [22] who reported different levels of expression of miR17-92 members in CLL patients expressing mutated TP53.

## Conclusion

Overexpression of all members of the cluster in Egyptian CLL patients was detected. Significant relations between the miR17-92 cluster and several parameters in CLL patients were demonstrated. MiR18a, miR19b-1, and miR92a-1 have an adverse prognostic value while miR17 can be considered a good prognostic marker in Egyptian CLL patients. High expression of miR19a is associated with better OS.

## Data Availability

All data generated or analyzed during this study are included in the published article.

## Abbreviations

CLL: Chronic lymphocytic leukemia
LDT: Lymphocyte doubling time
ALC: Absolute lymphocytic count
β2M: β2 Microglobulin
miRs: MicroRNAs
iwCLL: International Workshop on CLL
CBC: Complete blood counts
FISH: Fluorescence in situ hybridization
cDNA: Complementary DNA
CT: Computerized tomography
CR: Complete remission
PR: Partial remission
OS: Overall survival
